# Bifidobacterium bifidum postbiotics prevent Salmonella Pullorum infection in chickens by modulating pyroptosis and enhancing gut health

**DOI:** 10.1016/j.psj.2025.104968

**Published:** 2025-03-01

**Authors:** Yuhao Chen, Fuqiang Zhu, Guobi Yu, Nana Peng, Xinying Li, Meng Ge, Lei Yang, Wei Dong

**Affiliations:** College of Veterinary Medicine, Hunan Agricultural University, Changsha 410125, China

**Keywords:** Postbiotics, Bifidobacterium bifidum, Chicken, Intestinal health, Pyroptosis

## Abstract

The overuse of antibiotics in poultry farming has led to the emergence of multidrug-resistant pathogens, posing severe threats to animal health and public safety. *Salmonella Pullorum* (*S. Pullorum*), a host-specific pathogen targeting poultry, causes high mortality in chicks and disrupts intestinal health. This study evaluated the protective effects of *Bifidobacterium bifidum* postbiotics (BbP) against *S. Pullorum* infection, focusing on their mechanisms in regulating pyroptosis, restoring intestinal barrier function, and modulating gut microbiota. Both *in vivo* (chickens challenged with *S. Pullorum*) and *in vitro* (chicken small intestinal epithelial cells, CSIEC) models were used to assess the effects of BbP and its components (bacterial lysates or metabolites). Results showed that BbP significantly improved growth performance in infected chickens, reducing mortality from 66.66 % to 8.33 %. BbP effectively suppressed the expression of pyroptosis-related proteins, including apoptosis-associated speck-like protein containing a CARD (ASC), Caspase-1 (cysteine-aspartic acid protease-1), and Gasdermin D N-terminal (GSDMD-N), and reduced inflammatory cytokines, including interleukin-1β (IL-1β) and interleukin-8 (IL-8), while increasing anti-inflammatory cytokines, such as interleukin-10 (IL-10) and interleukin-4 (IL-4), thereby mitigating inflammation. Furthermore, BbP restored intestinal barrier function by upregulating the expression of tight junction proteins, including zonula occludens-1 (ZO-1), Occludin, and Claudin-1. The cecal microbiota diversity was also improved by BbP, with a decrease in the abundance of harmful bacteria (e.g., *Escherichia-Shigella*) and an enrichment of beneficial bacteria (e.g., *Lactobacillus and Ruminococcus*). These findings demonstrate that BbP provides significant protection against *S. Pullorum* infection by modulating pyroptosis, protecting the intestinal barrier, and restoring microbial balance. As an effective antibiotic alternative, BbP shows promise for the prevention and control of S. Pullorum infections in poultry farming.

## Introduction

In the past, antibiotics were extensively used as feed additives in poultry farming to promote growth and prevent bacterial infections (Wang et al., 2020). However, prolonged exposure of pathogenic microorganisms to prophylactic antibiotics has led to the rapid spread of antibiotic-resistant strains in poultry production facilities. These resistant pathogens can be transmitted to humans through eggs and chicken meat, posing significant threats to both the poultry industry and public biosecurity (Castro-Vargas et al., 2020). To address the issue of antibiotic resistance, the European Union banned antibiotics in animal feed in 2006, followed by the United States in 2017, and China fully implemented a similar policy in 2020. While these policies are crucial for safeguarding public health, they have also resulted in an increase in bacterial diseases and slower growth rates in poultry production (Fonseca et al., 2024).

*Salmonella Pullorum* (*S. Pullorum*), a host-specific pathogen that primarily infects poultry, induces the aggregation of inflammasome complexes during host cell infection, leading to the activation of apoptosis-associated speck-like protein containing a CARD (ASC) and recruitment of Caspase-1 (Kesavardhana et al., 2020). Activated inflammasomes convert inactive pro-Caspase-1 into its active form through autocleavage, enabling Caspase-1 to cleave its substrates. In addition to cleaving Gasdermin D (GSDMD), activated Caspase-1 processes pro-interleukin-1β (pro-IL-1β) into its biologically active form, thereby promoting inflammatory responses. During pyroptosis, the N-terminal domain of Gasdermin family proteins oligomerizes on the cell membrane to form 10–20 nm diameter pores, ultimately causing membrane rupture, release of intracellular contents, nuclear condensation, DNA fragmentation, and massive release of inflammatory factors. This inflammatory response results in cell death (Vasudevan et al., 2023).

Early-stage *S. Pullorum* infection causes severe damage to the intestinal tract and microbiota of chicks, subsequently spreading to host tissues and organs, leading to systemic lesions. Mortality rates are highest in chicks aged 2–3 weeks (Liu et al., 2022). Therefore, identifying a safe and effective antibiotic alternative to mitigate the effects of *S. Pullorum* infection is essential for the poultry industry.

In recent years, probiotics have demonstrated great potential in modulating the immune system and preventing intestinal diseases. Specifically, *Bifidobacterium* has been shown to regulate host immunity by enhancing intestinal vitality, inhibiting the invasion of intestinal pathogens, and maintaining intestinal microbiota homeostasis (Chen et al., 2021). However, probiotics need to reach sufficient colonization levels to effectively influence host immune or metabolic processes (Fang et al., 2024), while challenges remain regarding their poor stress resistance and difficulties in storage and transportation (Tao et al., 2024). Unlike probiotics, postbiotics are defined as “preparations of inanimate microorganisms and/or their components that confer a health benefit on the host” (Salminen et al., 2021). Postbiotics include bacterial cell components and their metabolites, which can effectively regulate host immunity, metabolism, and maintain intestinal microbiota balance (Lewis et al., 2015). Compared to probiotics, postbiotics exhibit longer shelf life, greater stability, and more convenient storage and transportation. Furthermore, postbiotics offer broader application potential, with reusable production processes and precise dosage control (Kavita et al., 2024). More importantly, postbiotics are safer than live probiotics as they avoid the accumulation of antibiotic resistance genes and virulence factors (Mehta et al., 2023).

This study aims to evaluate the effectiveness of *Bifidobacterium bifidum* postbiotics (BbP) in preventing *S. Pullorum* infection by establishing a chick infection model and a chicken small intestinal epithelial cell (CSIEC) infection model. Additionally, it investigates the mechanisms through which *S. Pullorum* induces pyroptosis in CSIEC and how BbP mitigates this process. Our research seeks to provide a viable antibiotic alternative for addressing *S. Pullorum*-associated challenges in poultry production.

## Materials and methods

### Bacterial strains and preparation of postbiotics

*Bifidobacterium bifidum* was inoculated into de Man, Rogosa, and Sharpe (MRS) broth supplemented with l-cysteine hydrochloride and incubated under anaerobic conditions at 37 °C for 36 h. When the bacterial count reached 1 × 10⁹ CFU/mL, a portion of the *Bifidobacterium bifidum* culture was collected as the probiotic control (Bb). The remaining *Bifidobacterium bifidum* culture was fully inactivated to prepare *Bifidobacterium bifidum* postbiotics (BbP). The inactivated culture was centrifuged to remove the supernatant, yielding postbiotics containing only inactivated *Bifidobacterium* cells (BbPbacterial). The remaining supernatant was filtered through a 0.22 μm membrane to obtain postbiotics containing only *Bifidobacterium* metabolites (BbPsupernatant).

### Animal experiment design

The animal experiments were carried out in accordance with the Guide for the Care and Use of Agricultural Animals in Research and Teaching, and approved by the Animal Ethics Committee of Hunan Agricultural University. Seventy-two 1-day-old Partridge Shank chicks (sex ratio 1:1) were purchased from Changsha Qiusheng Poultry Farm (Changsha, China). Throughout the experiment, the chicks were maintained at an ambient temperature of 30 ± 2 °C under a 12-hour light/dark cycle with ad libitum access to feed and water. The diet consisted of 521 starter feed for chicks (Charoen Pokphand Group). Initial body weights were recorded, and chicks were weighed every two days thereafter. The chicks were randomly divided into six groups as follows: the control group (normal water and feed without S. Pullorum challenge), SP group (normal water and feed with S. Pullorum challenge), BbP+SP group (1 mL of BbP daily via oral gavage with S. Pullorum challenge), BbPbacterial+SP group (1 mL of BbPbacterial daily via oral gavage with S. Pullorum challenge), BbPsupernatant+SP group (1 mL of BbPsupernatant daily via oral gavage with S. Pullorum challenge), and Bb+SP group (1 mL of Bb daily via oral gavage with S. Pullorum challenge).

After 7 days, chicks in the SP, BbP+SP, BbPbacterial+SP, BbPsupernatant+SP, and Bb+SP groups were orally challenged with 0.1 mL of *Salmonella Pullorum* suspension (1 × 10⁸ CFU per chick). The control group was given an equivalent volume of physiological saline. Following the challenge, all groups continued receiving their respective treatments for an additional 7 days. Mortality was observed and recorded during this period. At the end of the experiment, four healthy chicks from each group were randomly selected for cardiac blood sampling and euthanasia.

### Sample collection and processing

After cardiac blood collection, the blood was transferred into 2 mL centrifuge tubes and stored overnight at 4°C. The samples were centrifuged at 3,500 × g for 15 min, and the serum was separated and transferred to 1.5 mL centrifuge tubes for storage at −80 °C. The heart, liver, and spleen were weighed and divided into two portions: one was fixed in pre-cooled 4 % paraformaldehyde for histopathological analysis, and the other was frozen at −80 °C for inflammatory marker detection.

Segments of 2–3 cm from the duodenum, jejunum, and ileum were collected and fixed in 4 % paraformaldehyde for intestinal histopathology. Additional 2–3 cm sections of the small intestine were collected, contents removed, and the tissues frozen at −80 °C for the detection of inflammatory markers and intestinal barrier function. The cecum was also collected, with one portion frozen at −80 °C for 16S rRNA metagenomic analysis and the other fixed in 4 % paraformaldehyde for pathological examination.

### Measurement of serum inflammatory cytokines by ELISA

The concentrations of inflammatory cytokines IL-1β, IL-8, IL-4, and IL-10 in the serum of chicks from each group were measured using chicken ELISA kits (Jiangsu Meimian Industrial Co., Ltd., China). All experiments were repeated three times to ensure the reliability and reproducibility of the results.

### RNA extraction and qPCR for mRNA expression analysis

A 0.1 g sample from each tissue was added to 1 mL of Trizol reagent in a 1.5 mL sterile Eppendorf tube and homogenized using a tissue grinder. Total RNA was extracted using the chloroform and isopropanol method, and RNA concentrations were measured with a Nano Photometer spectrophotometer (Implen GmbH, Germany). Subsequently, RNA was reverse-transcribed into cDNA using the TOROIVD® qRT Master Mix 2.0 (TOROIVD, Shanghai, China). The cDNA concentration was adjusted to 100 ng/mL for qPCR experiments.

Primers for the target genes were designed using Primer 5.0 software, and their specificity was verified using BLAST. The primer sequences for the target genes are shown in Table 1. Quantitative real-time PCR was performed using TOROGreen® qPCR Master Mix on the SLAN automated medical PCR analysis system. The relative mRNA expression levels were calculated using the 2^−ΔΔCT^ method. All experiments were repeated three times to ensure the reliability and reproducibility of the results.

**Table 1:** Primer Sequences.

Target genes	Forward primers (5′—3′)	Reverse primers (5′—3′)
β-actin	ACGTCGCACTGGATTTCGAG	TGTCAGCAATGCCAGGGTAC
SP	TCTAGCACTGAACTTGGCGA	TGTGTCGCCATTGTAGGTCA
IL-1β	ACTGGGCATCAAGGGCTA	GGTAGAAGATGAAGCGGGTC
IL-8	ATGAACGGCAAGCTTGGAGCTG	TCCAAGCACACCTCTCTTCCATCC
IL-10	GCGCTTCTACACAGATGAGGT	CGAACGTCTCCTTGATCTGC
TLR4	TGACCTACCCATCGGACACT	TGCCTGAGAGAGGTCAGGTT
Caspase-1	CTCTGACAGCACCTTCCTGG	GCATCTCAGGGTCGATGGAG
ZO-1	CTTCAGGTGTTTCTCTTCCTCCTC	CTGTGGTTTCATGGCTGGATC
Occludin	ACGGCAGCACCTACCTCAA	GGGCGAAGAAGCAGATGAG
Claudin-1	CATACTCCTGGGTCTGGTTGGT	GACACGCATCCGCATCTTCT

### Cecal microbiota 16S rRNA gene sequencing analysis

Total genomic DNA from cecal microbiota was extracted using the E.Z.N.A™ Mag-Bind Soil DNA Kit (Omega, M5635-02, USA) following the manufacturer's instructions. The 16S rRNA gene V3–V4 region was amplified using the forward primer (CCTACGGGNGGCWGCAG) and reverse primer (GACTACHVGGGTATCTAATCC) with 2 × Hieff® Robust PCR Master Mix (Yeasen, 10105ES03, China). The PCR reaction (30 µl) consisted of 2 µl microbial DNA (10 ng/µL), 1 µl forward primer (10 µM), 1 µl reverse primer (10 µM), and 26 µl master mix. The thermal cycling conditions were: initial denaturation at 95 °C for 3 min, followed by 5 cycles of denaturation at 95°C for 30 s, annealing at 45°C for 30 s, and extension at 72 °C for 30 s; then 20 cycles with annealing at 55 °C; and a final extension at 72 °C for 5 min. The PCR products were purified using Hieff NGS™ DNA Selection Beads (Yeasen, 10105ES03, China) to remove free primers and primer dimers. Purified products were sent to Sangon Biotech (Shanghai) for library construction using universal Illumina adaptors and indexes. Sequencing was performed on the Illumina MiSeq system (Illumina MiSeq, USA) according to the manufacturer's protocol. All experiments were repeated three times to ensure the reliability and reproducibility of the results.

### Effect of BbP on the viability of CSIEC by MTT assay

Postbiotics were diluted to different concentrations (0.25 %, 0.5 %, 1 %, 2 %, 3 %, 4 %, 5 %) using DMEM medium containing 10 % fetal bovine serum (FBS). CSIEC cells were seeded in 96-well plates and cultured in DMEM medium containing 10 % FBS until they reached 80 % confluency. Each well was then incubated with 100 μL of postbiotics at different concentrations, with 10 replicates for each concentration. After 10 h of incubation, 10 μL of MTT solution (5 mg/mL) was added to each well, followed by an additional 4 h of incubation. Subsequently, 100 μL of DMSO was added to each well and shaken for 10 min. The absorbance was measured at 490 nm using a microplate reader.

### Determination of the effect of BbP on the adhesion of *S. Pullorum* to CSIEC by plate counting and immunofluorescence

Chicken small intestinal epithelial cells (CSIEC) were harvested, and the cell suspension was counted using a Countstar Mira BF Automated Cell Analyzer. The cell suspension was then diluted to a density of 2 × 10⁵ cells/mL with fresh medium. A volume of 0.5 mL of the diluted cell suspension was added to each well of a 24-well plate, resulting in 1 × 10⁵ cells/well. The cells were cultured until they reached 80 % confluency to ensure consistent cell numbers across all experimental groups. The following assays were performed: exclusion, competition, and displacement tests, with a positive control group included. Each experiment was conducted in triplicate.

In the exclusion assay, cells were pretreated with 1 % postbiotics (500 μL) for 1 h, followed by infection with *S. Pullorum* at a multiplicity of infection (MOI) of 200 for another hour. In the competition assay, postbiotics and *S. Pullorum* were co-incubated for 1 h, and the mixture was then used to infect the cells for an additional hour. In the displacement assay, cells were first infected with *S. Pullorum* at an MOI of 200 for 1 h, followed by treatment with 1 % postbiotics (500 μL) for another hour.

After the assays, the cells were washed three times with phosphate-buffered saline (PBS) and treated with 100 μL of 0.25 % trypsin (purchased from Coolaber Biotech, Beijing) for 3 min to detach the cells. The reaction was terminated by adding 100 μL of cell culture medium. The cell suspension was collected and subjected to bacterial colony counting. Each experiment was repeated three times.

For immunofluorescence analysis, the pretreatment with BbP and *S. Pullorum* was identical to the steps described above for the plate count assay. Following these treatments, the cells were fixed with 4 % paraformaldehyde for 15 min and blocked with immunofluorescence blocking buffer (purchased from Coolaber Biotech, Beijing) at 37 °C for 30 min. Subsequently, the cells were incubated with a rabbit primary antibody against *S. Pullorum* at 37 °C for 30 min, followed by incubation with a goat anti-rabbit secondary antibody conjugated with DyLight488 fluorescence (Abbkine, A23220) at 37 °C for another 30 min. Finally, the cells were observed under a fluorescence microscope. All experiments were repeated three times to ensure the reliability and reproducibility of the results.

### Effect of BbP on pyroptosis in chicken small intestinal epithelial cells induced by Salmonella Pullorum

Chicken small intestinal epithelial cells (CSIEC) were seeded into 12-well plates and cultured until 80 % confluency. The cells were incubated with 1 % *Bifidobacterium bifidum* postbiotics (BbP) for 10 h, followed by infection with *S. Pullorum* at a MOI of 200. At 3, 8, 13, and 20 h post-infection, cells were harvested, and total protein was extracted. Equal amounts of protein (20 μg) were separated on a 12 % SDS-PAGE gel and transferred to a PVDF membrane.

The membrane was blocked with 5 % skimmed milk and incubated overnight (16 h) at 4 °C with primary antibodies against ASC, Caspase-1, GSDMD-N, and β-actin (purchased from Immunoway Biotech, China). After washing, the membrane was incubated with horseradish peroxidase (HRP)-conjugated goat anti-rabbit secondary antibody (Abbkine, A21020) at room temperature. Finally, the membrane was treated with enhanced chemiluminescence (ECL) detection reagent (Abbkine, ATWD14081), and signals were visualized using a chemiluminescence imaging system (Bio-Rad). Band intensities were analyzed using Image Lab software, with β-actin serving as the internal control for normalization. After Western blotting, band intensities were quantified using Image Lab 5.0 software (Bio-Rad), with β-actin as the internal reference for normalization. All experiments were repeated three times to ensure the reliability and reproducibility of the results.

### Statistical analysis

Data were analyzed using IBM SPSS software (version 27.0, Chicago, IL), Image Lab 5.0, and GraphPad Prism software (version 8.0.2.2, Chicago, IL). A p-value of <0.05 was considered statistically significant.

## Results

### Bifidobacterium bifidum postbiotics improve growth performance in chicks

**Fig. 1:**
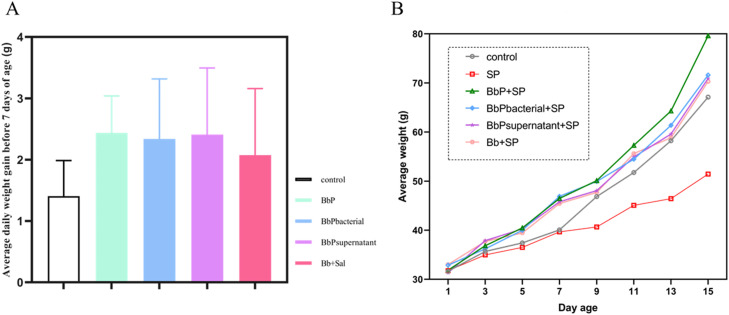
Effect of *Bifidobacterium bifidum* Postbiotics on the Growth Performance of Chicks. (A) Effect of postbiotics on the average body weight of chicks; (B) Effect of postbiotics on the average daily weight gain of chicks before 7 days of age.

Before 7 days of age, there was no significant difference in average body weight between the Control and SP groups (*P* > 0.05). However, the BbP+SP, BbPbacterial+SP, BbPsupernatant+SP, and Bb+SP groups showed significantly higher average body weight (Fig. 1A) and daily weight gain (Fig. 1B) compared to the Control group (*P* < 0.05), indicating that Bifidobacterium bifidum postbiotics promoted chick growth. Between 7 and 15 days of age, S. Pullorum infection significantly reduced body weight in the SP group, while all treatment groups maintained significantly higher body weights than the SP group (*P* < 0.05). Among them, the BbP+SP group showed the greatest weight gain (Fig. 1A). In summary, S. Pullorum infection inhibited chick growth, but Bifidobacterium bifidum postbiotics alleviated this effect and improved growth both before and after infection, with the BbP+SP group showing the most pronounced results.

### Bifidobacterium bifidum postbiotics reduce mortality in salmonella Pullorum-infected chickens

During the experimental period, the number of chick deaths in the SP group significantly increased following Salmonella Pullorum infection, as shown in Table 2. A total of 8 deaths were observed, resulting in a mortality rate of 66.66 %. In contrast, treatment with *Bifidobacterium bifidum* postbiotics significantly reduced the mortality rate in all experimental groups. Specifically, the BbP+SP group showed the most effective protection, with only 1 death and a mortality rate of 8.33 %. The BbPbacterial+SP group recorded 2 deaths (16.66 %), while the BbPsupernatant+SP and Bb+SP groups each recorded 3 deaths (25 %).

**Table 2:** Mortality rate of chicks infected with *Salmonella Pullorum*.

Group	Number of deaths	Mortality rate (%)
control	0	0
SP	8	66.66
BbP+SP	1	8.33
BbPbacterial+SP	2	16.66
BbPsupeinatant+SP	3	25
Bb+SP	3	25

These results indicate that *Bifidobacterium bifidum* postbiotics significantly reduced the mortality rate caused by *Salmonella Pullorum* infection in chicks, with the BbP+SP group showing the most pronounced protective effect.

### Bifidobacterium bifidum Postbiotics reduce liver and intestinal damage in chicks caused by Salmonella Pullorum fowl dysentery

**Fig. 2:**
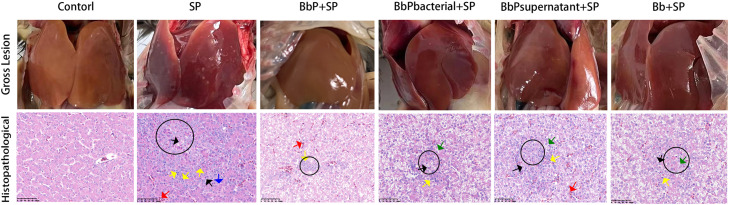
Gross liver lesions in chicks. Red arrows indicate sinusoidal congestion; blue arrows indicate fatty degeneration of hepatocytes; green arrows indicate vacuolar degeneration; black arrows indicate hepatocyte necrosis; yellow arrows indicate inflammatory cell infiltration; black circles indicate necrotic foci. Scale bar = 200 μm .

***Gross and Histopathological Observations of Chicken Liver.*** Gross examination revealed that the liver surface in the Control group was smooth and free of visible lesions. HE staining showed normal liver histology, with well-organized hepatic cords, clear sinusoids, and healthy hepatocytes with evenly stained cytoplasm and centrally located nuclei (Fig. 2). In contrast, the SP group exhibited severe pathological changes, including numerous white nodules on the liver surface, disorganized hepatic cords, sinusoidal dilation with congestion, fatty degeneration, vacuolar degeneration, and focal necrotic areas surrounded by significant inflammatory cell infiltration.

In the treatment groups, the white nodules on the liver surface were significantly reduced. HE staining showed that BbPbacterial+SP, BbPostbiotic+SP, and Bb+SP groups had alleviated sinusoidal congestion, reduced vacuolar degeneration, smaller necrotic areas, and decreased inflammatory cell infiltration. Among all groups, the BbP+SP group showed the most significant protective effects, with nearly normal liver structure. Gross examination revealed no visible lesions, and HE staining showed only mild inflammatory cell infiltration without evident necrosis or severe hepatocyte damage.

**Fig. 3:**
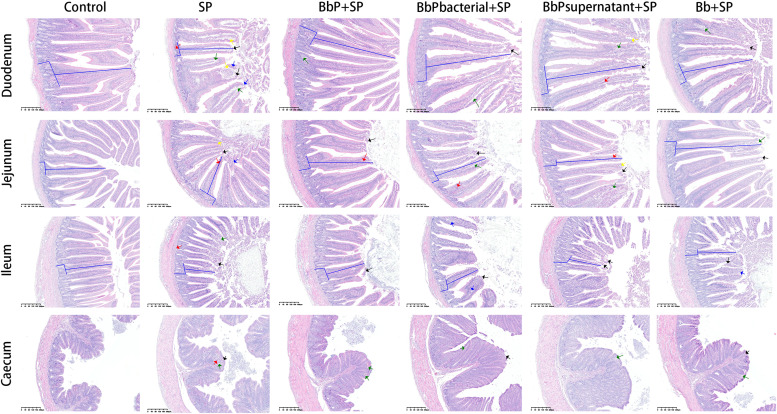
Representative histopathological images of the duodenum, jejunum, ileum, and cecum from different experimental groups (HE staining). Green arrows indicate lamina propria edema; blue arrows indicate vacuolar degeneration of epithelial cells; black arrows indicate epithelial cell necrosis and detachment; yellow arrows indicate inflammatory cell infiltration; red arrows indicate capillary congestion. Scale bar = 200 μm.

***Pathological Analysis of Intestinal Lesions in Chicks.*** Based on HE-stained sections of the duodenum, jejunum, ileum, and cecum (Fig. 3), lesion severity was scored according to the grading criteria shown in Table 3. The Control group exhibited no lesions, with intact tissue structure and a healthy state (score = 0). In contrast, the SP group had the highest lesion scores (16–19 points), characterized by severe capillary congestion, extensive epithelial necrosis and detachment, lamina propria edema, and inflammatory cell infiltration, indicating significant tissue damage. Postbiotic treatments significantly reduced lesion scores compared to the SP group. Among the treatment groups, BbP+SP exhibited the lowest scores (1–4 points), with the cecum nearly restored to normal and minimal lesions in other intestinal segments. BbPbacterial+SP and BbPsupernatant+SP also alleviated tissue damage but were less effective than BbP+SP, while Bb+SP showed the weakest improvement.

**Table 3:** Criteria for rating intestinal tissue lesions.

Pathological Feature	No Lesion (0 points)	Mild Lesion (1 point)	Moderate Lesion (2 points)	Severe Lesion (3 points)
Capillary Congestion	No congestion	Slight congestion	Moderate congestion	Severe congestion
Lamina Propria Edema	No edema	Mild edema	Moderate edema	Severe edema
Epithelial Necrosis	No necrosis	Few necrotic cells	Moderate epithelial necrosis	Extensive epithelial necrosis
Villus Detachment	No detachment	Villus structure intact with minor damage	Moderate detachment of villus tips	Severe villus detachment or widespread shedding
Inflammatory Cell Infiltration	No infiltration	Slight infiltration	Moderate infiltration	Severe infiltration surrounding necrotic regions
Vacuolar Degeneration	No degeneration	Few cells with vacuolar changes	Moderate vacuolar degeneration in localized regions	Widespread vacuolar degeneration

**Fig. 4:**
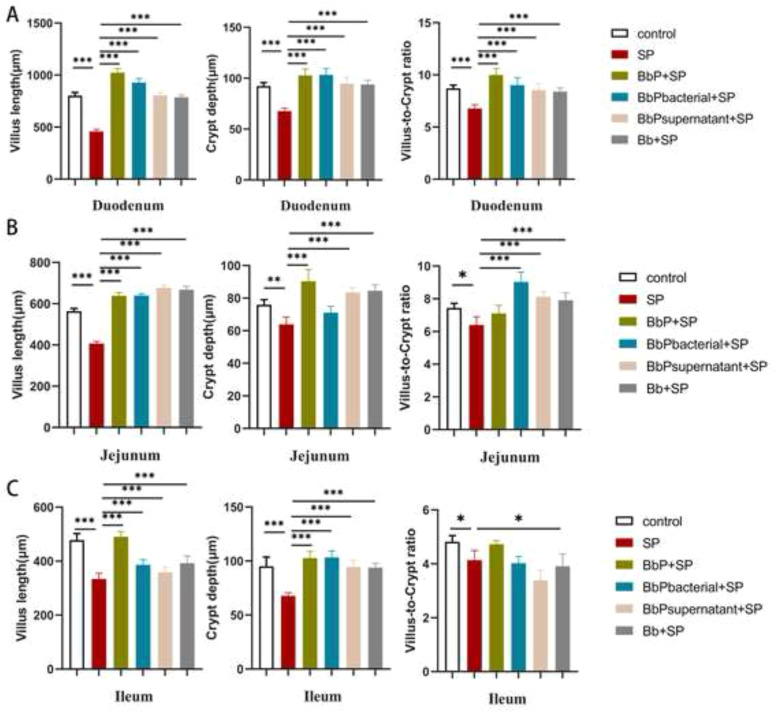
Effects of different treatments on intestinal villus length, crypt depth, and villus-to-crypt ratio in the duodenum, jejunum, and ileum of chicks. (A)duodenum, (B)jejunum, (C) ileum. Data are presented as mean ± SEM. *P < 0.05, **P < 0.01, ***P < 0.001,compared to the SP group.

The histopathological findings were consistent with changes in intestinal morphology (Fig. 4). In the SP group, villus length was significantly reduced, crypt depth increased, and the villus-to-crypt ratio decreased in the duodenum, jejunum, and ileum compared to the Control group (*P* < 0.001), reflecting severe structural damage caused by Salmonella Pullorum. Postbiotic treatments significantly reversed these changes, with BbP+SP showing the greatest improvement, restoring villus length and the villus-to-crypt ratio to near-Control levels. BbPbacterial+SP and BbPsupernatant+SP also showed protective effects, while Bb+SP had a weaker impact.

In summary, Bifidobacterium bifidum postbiotics (BbP) effectively alleviated intestinal lesions and restored intestinal morphology, demonstrating superior protective effects compared to other treatments. Fig. 3. HE-stained sections of the duodenum, jejunum, and ileum. The green arrows indicate lamina propria edema, the blue arrows indicate vacuolar degeneration of cells, the black arrows indicate epithelial cell necrosis and detachment, the yellow arrows indicate inflammatory cell infiltration, and the red arrows indicate capillary congestion.

### Bifidobacterium bifidum postbiotics modulate serum inflammatory cytokines in Salmonella Pullorum-infected chickens

**Fig. 5:**
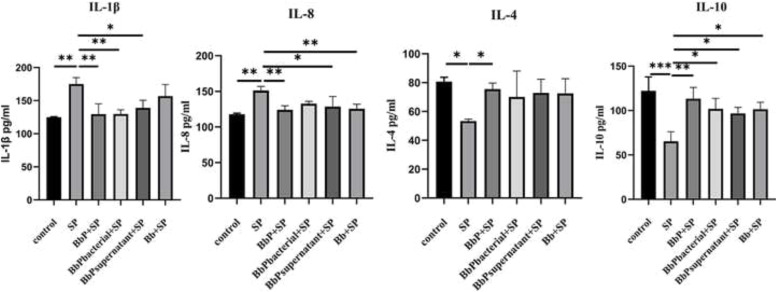
Levels of inflammatory cytokines in chick serum. Data are presented as Mean ± SD and analyzed by one-way ANOVA Tukey test. * *P* < 0.05, ** *P* < 0.01, *** and **** *P* < 0.001.

In the serum of chicks, the SP group exhibited significantly higher IL-1β and IL-8 concentrations and lower IL-4 and IL-10 levels compared to the Control group (*P* < 0.01, Fig. 5). Postbiotic treatments significantly reduced IL-1β levels in all treatment groups (*P* < 0.05), with the BbP+SP group showing the strongest inhibitory effect. Similarly, IL-8 concentrations were significantly lower in the BbP+SP and BbPsupernatant+SP groups compared to the SP group (*P* < 0.05), while no significant changes were observed in the BbPbacterial+SP and Bb+SP groups (*P* > 0.05). In contrast, postbiotic treatments significantly increased IL-10 levels in all groups (*P* < 0.01), with the BbP+SP group exhibiting the most pronounced upregulation (*P* < 0.05). IL-4 levels were also significantly elevated in the BbP+SP and BbPsupernatant+SP groups (*P* < 0.05) but remained unchanged in the BbPbacterial+SP and Bb+SP groups (*P* > 0.05).

In summary, B. bifidum postbiotics effectively reduced pro-inflammatory cytokines (IL-1β and IL-8) while enhancing anti-inflammatory cytokines (IL-10 and IL-4), with the BbP+SP group demonstrating the most significant regulatory effects.

### Bifidobacterium bifidum postbiotics reduce Salmonella Pullorum colonization in chick tissues

**Fig. 6:**
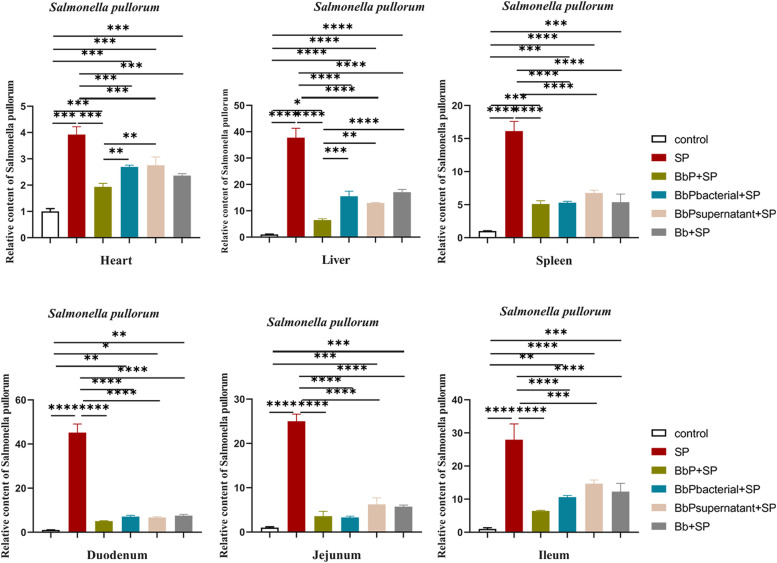
Colonization levels of Salmonella Pullorum in the heart, liver, spleen, duodenum, jejunum, and ileum of chicks. Data are presented as Mean ± SD and analyzed by one-way ANOVA Tukey test. * *P* < 0.05, ** *P* < 0.01, *** and **** *P* < 0.001.

The relative abundance of *Salmonella Pullorum* in the heart, liver, spleen, duodenum, jejunum, and ileum was significantly higher in the SP group compared to the Control group (*P* < 0.001,Fig. 6). Compared to the SP group, the colonization levels of *S. Pullorum* in all six tissues were significantly reduced in the BbP+SP, BbPbacterial+SP, and BbPsupernatant+SP groups (*P* < 0.001 or *P* < 0.01). However, in the Bb+SP group, although the colonization levels of *S. Pullorum* decreased, statistical significance was not observed in certain tissues, such as the heart and jejunum.

Furthermore, the BbP+SP group demonstrated the most pronounced inhibitory effect across all tissues, with *S. Pullorum* colonization levels being comparable to or lower than those in the other treatment groups.

### Bifidobacterium bifidum Postbiotics suppress pro-inflammatory cytokine and receptor expression in organs and intestines of Salmonella Pullorum-infected chickens

**Fig. 7:**
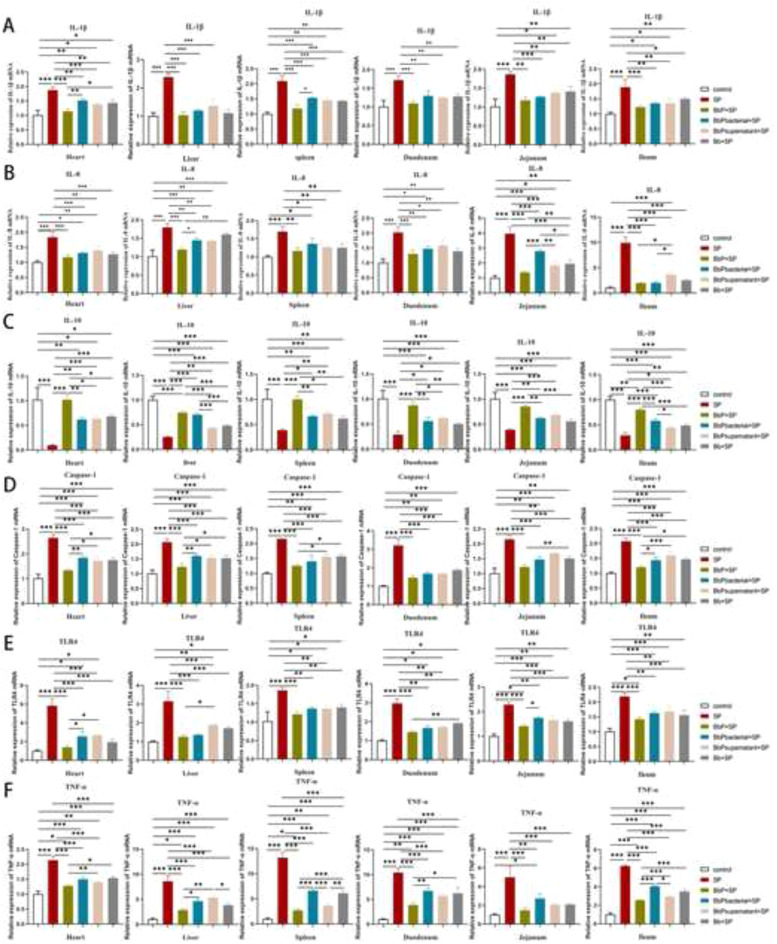
Relative mRNA expression levels of inflammatory factors in the heart, liver, spleen, duodenum, jejunum, and ileum of chicks. (A) IL-1β; (B) IL-8; (C) IL-10; (D) Caspase-1; (E) TLR4; (F) TNF-α. Data are presented as Mean ±SD and analyzed by one-way ANOVA Tukey test. * *P* < 0.05, ** *P* < 0.01, *** and **** *P* < 0.001.

In the heart, liver, spleen, and small intestine of chicks, S. Pullorum infection significantly upregulated the mRNA expression of pro-inflammatory cytokines (IL-1β, IL-8, TNF-α), inflammasome components (Caspase-1), and the pattern-recognition receptor TLR4 (all *P* < 0.01,Fig. 7). Conversely, the anti-inflammatory cytokine IL-10 was markedly downregulated (*P* < 0.01). Treatment with Bifidobacterium bifidum postbiotics (BbP) significantly reversed these effects. Compared to the S. Pullorum (SP) group: Expression of *IL-1β, IL-8, TNF-α, Caspase-1*, and *TLR4* was reduced by 40–65 % (*P* < 0.05), with the strongest inhibition observed in the BbP+SP group; *IL-10* expression increased by 2.1-fold (*P* < 0.01), restoring immune balance. These results demonstrate that postbiotics effectively suppress systemic pro-inflammatory responses by downregulating inflammasome activation (*Caspase-1*), cytokine production (*IL-1β, IL-8, TNF-α*), and receptor signaling (*TLR4*), while enhancing anti-inflammatory pathways (*IL-10*).

### Bifidobacterium bifidum postbiotics enhance expression of intestinal barrier proteins in salmonella Pullorum-infected chickens

**Fig. 8:**
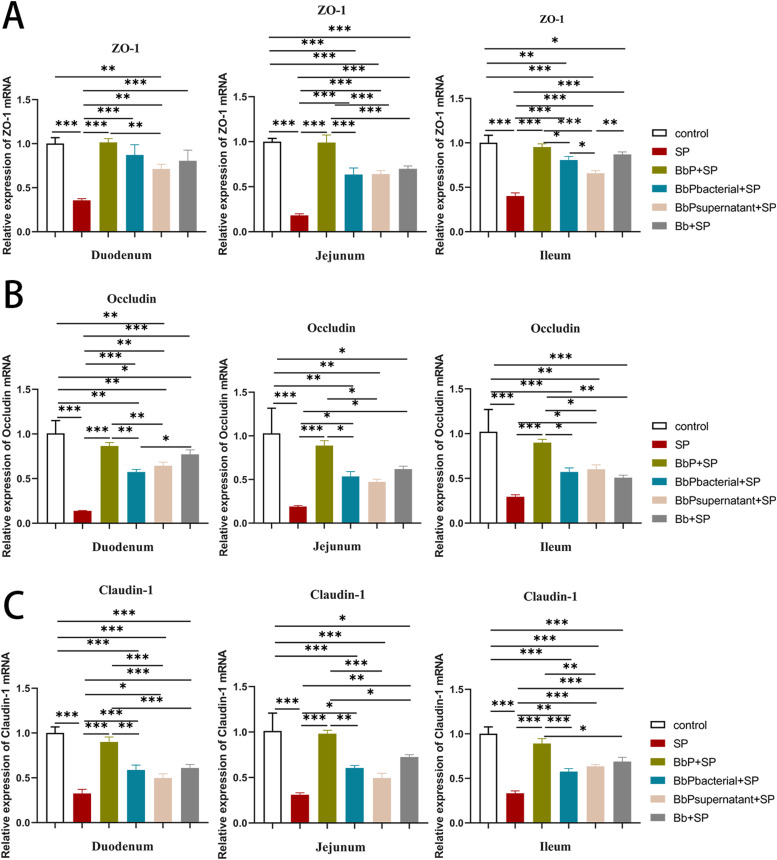
Relative mRNA expression levels of intestinal barrier proteins in the small intestine of chicks. (A) ZO-1; (B) Occludin; (C) Claudin-1. Data are presented as Mean ± SD and analyzed by one-way ANOVA Tukey test. * *P* < 0.05, ** *P* < 0.01, *** and **** *P* < 0.001.

In the duodenum, jejunum, and ileum, the relative mRNA expression levels of ZO-1, Occludin, and Claudin-1 were significantly lower in the SP group than in the Control group (*P* < 0.01, Fig. 8). These results suggest that *Salmonella Pullorum* infection suppressed the expression of tight junction proteins, which may lead to impaired intestinal barrier function.

Compared to the SP group, the mRNA expression levels of ZO-1, Occludin, and Claudin-1 were significantly increased in the BbP+SP, BbPbacterial+SP, BbPostbiotic+SP, and Bb+SP groups (*P* < 0.05 or *P* < 0.01). Among these, the BbP+SP group exhibited the most pronounced recovery, with expression levels approaching those of the Control group.

In conclusion, *Salmonella Pullorum* infection significantly suppressed the expression of tight junction proteins (ZO-1, Occludin, and Claudin-1), potentially causing intestinal barrier damage. However, *Bifidobacterium bifidum* postbiotics and their related treatments significantly increased the expression levels of tight junction proteins, alleviating the damage to intestinal barrier function caused by infection, with the BbP+SP group demonstrating the most effective protective effect.

### Bifidobacterium bifidum postbiotics restore Cecal Microbiota diversity in Salmonella Pullorum-infected chickens

**Fig. 9:**
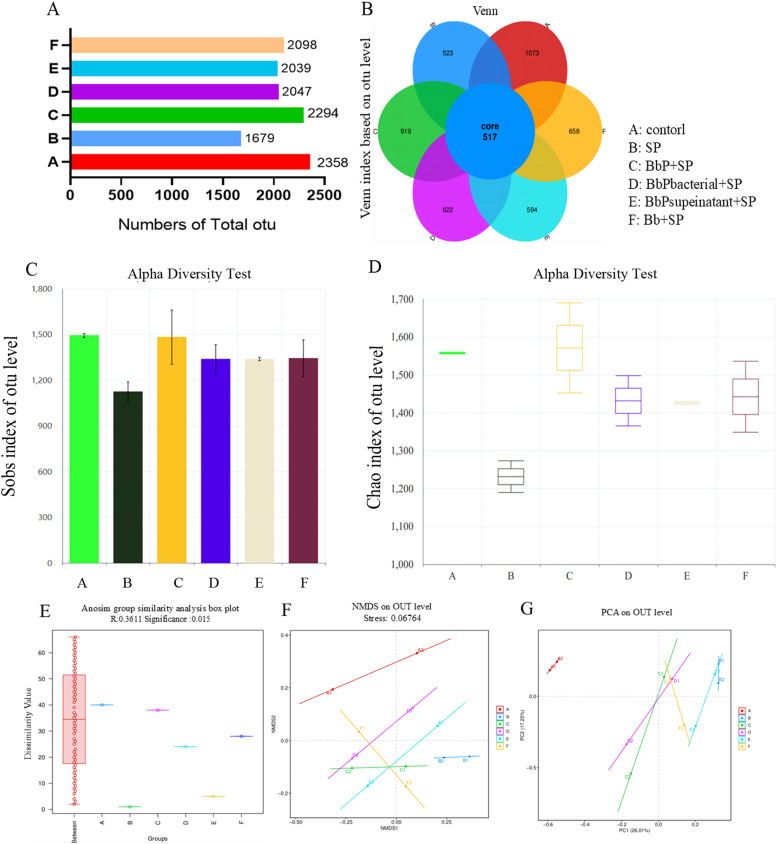
Effect of postbiotics on the OTU counts and alpha diversity of cecal microbiota in chicks. (A) Numbers of total OTUs; (B) OTU-based Venn maps of species distribution; (C) Alpha diversity based on the Sobs index; (D) Alpha diversity based on the Chao index; (E) Bray-Curtis distance at the OTU level; (F) Unweighted UniFrac distance at the OTU level; (G) PCA analysis at the OTU level.

After OTU clustering of non-redundant sequences (excluding singletons) at 97 % similarity, the total OTU count was 2,358 in the Control group, whereas the BbP+SP, BbPbacterial+SP, BbPsupernatant+SP, and Bb+SP groups had total OTU counts of 2,294, 2,047, 2,039, and 2,098, respectively. In contrast, the SP group had the lowest total OTU count at 1,679 (Fig. 9A). The Venn diagram revealed that the SP group had the lowest number of unique OTUs, while the other groups showed increased unique OTU counts, with the Control group having the highest number, followed by the BbP+SP group (Fig. 9B). In the α-diversity analysis based on the Sobs and Chao indices, all treatment groups (BbP+SP, BbPbacterial+SP, BbPsupernatant+SP, and Bb+SP) exhibited an increasing trend compared to the SP group, with the BbP+SP group showing the most pronounced improvement (Fig. 9C, D). Anosim analysis showed significant intergroup differences, with an R-value of 0.3611 for the SP group (Fig. 9E). Additionally, the β-diversity, as visualized by NMDS and PCA scatter plots, demonstrated a significant shift in the SP group compared to the other groups. In contrast, the BbP+SP, BbPbacterial+SP, BbPsupernatant+SP, and Bb+SP groups showed a trend toward convergence with the Control group (Fig. 9F, G). Bifidobacterium bifidum Postbiotics Modulate Dominant Cecal Microbiota in Salmonella Pullorum-Infected Chickens

**Fig. 10:**
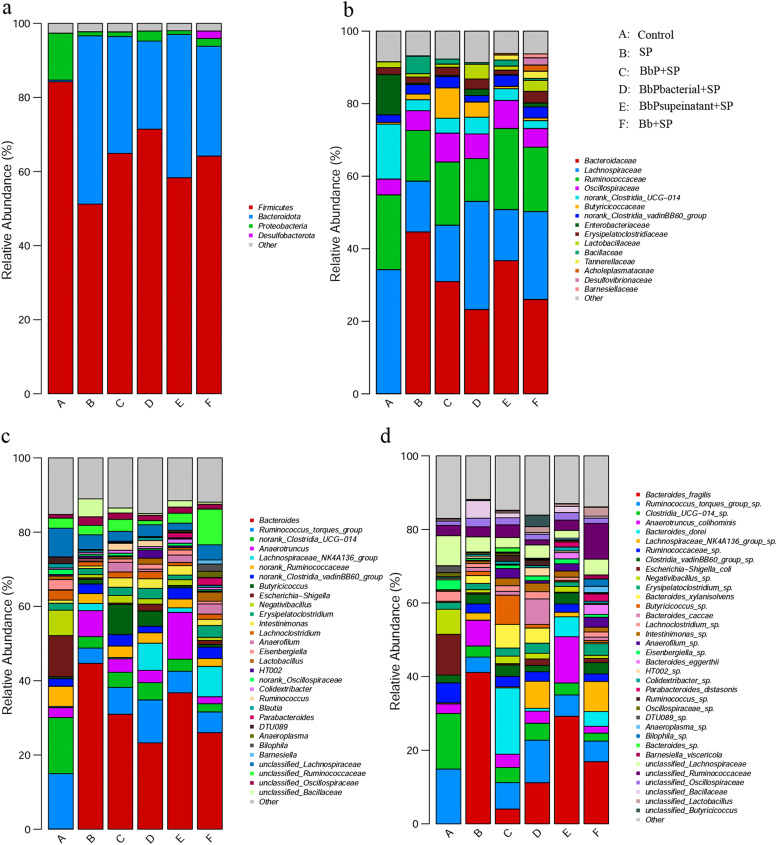
Effect of BbP on the relative abundance of microbial dominant species in the chick cecum.(a) Relative abundance analysis at the phylum level; (b) Relative abundance analysis at the family level; (c) Relative abundance analysis at the genus level; (d) Relative abundance analysis at the species level.

At the phylum level (Fig. 10A), the gut microbiota composition showed significant differences among the experimental groups. In the Control group, the gut microbiota was primarily composed of *Firmicutes* and *Bacteroidota*, with *Firmicutes* being the most dominant. In contrast, the *Salmonella*-infected SP group exhibited a significant reduction in *Firmicutes* abundance and a marked increase in the relative abundance of *Bacteroidota* and *Proteobacteria*, indicating severe disruption of gut microbiota structure caused by *Salmonella* infection. Compared to the SP group, the BbP+SP, BbPbacterial+SP, BbPsupernatant+SP, and Bb+SP groups showed varying degrees of recovery in *Firmicutes* abundance and a significant reduction in *Proteobacteria* levels, with the BbP+SP group demonstrating the most pronounced recovery, resulting in a microbial composition close to that of the Control group.

At the family level (Fig. 10B), the Control group was dominated by *Lachnospiraceae* and *Ruminococcaceae*. However, in the SP group, the relative abundance of *Desulfovibrionaceae* and *Enterobacteriaceae* significantly increased, further indicating the severe dysbiosis caused by *Salmonella* infection. Postbiotic treatments effectively restored the relative abundance of *Lachnospiraceae* and *Ruminococcaceae* in all experimental groups, with the BbP+SP group showing the most significant recovery, closely resembling the Control group.

At the genus level (Fig. 10C), *Escherichia-Shigella* was significantly increased in the SP group, while beneficial genera such as *Lactobacillus* and *Bacteroides* were significantly reduced. Compared to the SP group, the BbP+SP group significantly decreased the abundance of *Escherichia-Shigella* and increased the relative abundance of *Lactobacillus* and *Ruminococcus*, showing a more effective recovery than the BbPbacterial+SP and BbPsupernatant+SP groups.

At the species level (Fig. 10D), pathogenic bacteria such as *Bacteroides fragilis* were significantly enriched in the SP group, while beneficial bacteria such as *Ruminococcus torques group* were markedly reduced. In contrast, the BbP+SP group significantly inhibited the proliferation of pathogenic bacteria and promoted the recovery of beneficial gut microbiota, demonstrating a more pronounced effect than other treatment groups.

### BbP enhances cell viability, inhibits S. Pullorum adhesion, and suppression of pyroptosis in chicken small intestinal epithelial cells (CSIEC)

**Fig. 11:**
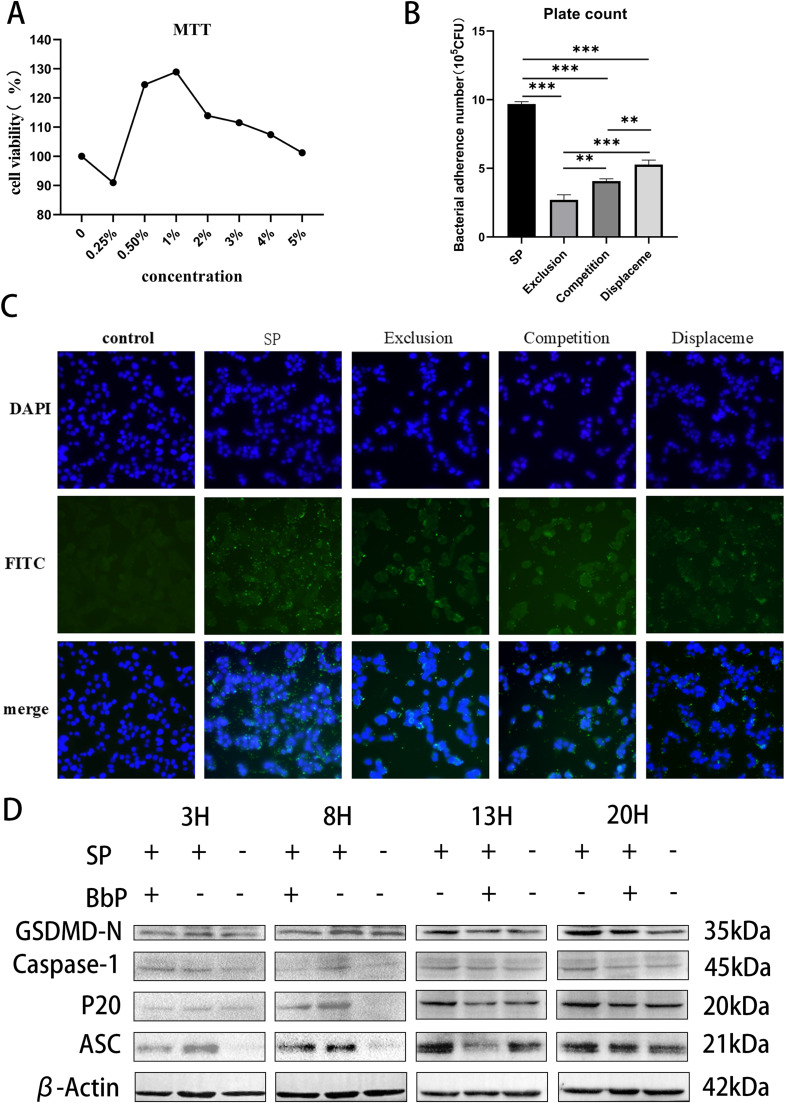
Effect of postbiotics on chicken small intestinal epithelial cells (CSIEC).(A) Cell viability of CSIEC under different postbiotic concentrations measured by MTT assay. (B) Bacterial adhesion to CSIEC under three intervention strategies: Exclusion, Competition, and Displacement. (C) Fluorescence microscopy showing bacterial adhesion to CSIEC under different intervention strategies (DAPI staining for cell nuclei in blue, FITC staining for bacteria in green). (D) Expression of pyroptosis-related proteins (ASC, Caspase-1, P20, GSDMD-N, and β-actin) in CSIEC at different time points (3 h, 8 h, 13 h, 20 h) detected by Western blotting. Data are presented as Mean ±SD and analyzed by one-way ANOVA Tukey test. ** *P* < 0.01, *** and **** *P* < 0.001.

The results of the MTT assay for CSIEC viability (Fig. 11A) showed that low concentrations (0.25 %−1 %) significantly increased cell viability, with the 1 % concentration group reaching a peak viability of approximately 120 %, which was significantly higher than the control group (0 % concentration). However, as the concentration increased to 2 %−5 %, cell viability began to decline, with the 5 % concentration group reducing viability to approximately 90 %. These results suggest that low concentrations of postbiotics promote cell viability, while higher concentrations may exert inhibitory effects on cells.

The effects of the three intervention strategies (Exclusion, Competition, and Displacement) of BbP on bacterial adhesion in the SP group were compared using the plate counting method (Fig. 11B). The SP group exhibited significantly higher bacterial adhesion compared to the intervention groups (*P* < 0.01), as indicated by a higher colony-forming unit (CFU) count. Among the intervention groups, the Exclusion group demonstrated the lowest bacterial adhesion, followed by the Competition and Displacement groups (*P* < 0.01). The significant inhibitory effect observed in the Exclusion group suggests that the exclusion mechanism effectively reduces bacterial adhesion to cells.

Fluorescence microscopy further verified the inhibitory effects of different intervention strategies on bacterial adhesion (Fig. 11C). In the SP group, abundant green fluorescence signals (with DAPI staining marking cell nuclei in blue and FITC staining marking adhered bacteria in green) indicated a high level of bacterial adhesion. Conversely, the Exclusion, Competition, and Displacement groups exhibited significantly weaker green fluorescence signals, with the Exclusion group showing the weakest fluorescence intensity, consistent with the plate counting results. This indicates that the exclusion mechanism is the most effective in inhibiting bacterial adhesion.

To investigate the effect of BbP on pyroptosis-related signaling pathways, Western blotting was performed to detect the expression levels of GSDMD-N, Caspase-1, P20, ASC, and β-Actin proteins at different time points (3 h, 8 h, 13 h, 20 h) (Fig. 11D). Gray value analysis further validated the suppression of pyroptosis by BbP, with significant reductions in ASC, Caspase-1, and GSDMD-N expression (**Supplementary Figure. S1**). At the early stages of infection (3 h), ASC protein (apoptosis-associated speck-like protein containing a CARD) expression in the SP group began to increase significantly (*P* < 0.05) and remained elevated at 20 h, while BbP treatment groups showed a clear downregulation trend compared to the SP group.

For Caspase-1, the SP group showed a noticeable upregulation only at 8 h compared to the control group, whereas the BbP group exhibited a downregulation trend compared to the SP group. The activated fragment of Caspase-1, P20, increased in a time-dependent manner, with the highest expression observed in the SP group, followed by the BbP treatment group.

The expression of the pyroptosis execution protein GSDMD-N gradually increased over time, showing a significant increase at 8 h and remaining elevated at 13 h and 20 h in the SP group. In contrast, GSDMD-N expression was consistently lower in the BbP treatment group than in the SP group. These results indicate that BbP effectively inhibits the initiation of the pyroptosis pathway by downregulating ASC expression, suppressing Caspase-1 activation, and preventing GSDMD cleavage.

## Conclusion and discussion

This study constructed animal models of Salmonella Pullorum infection in chicks and an in vitro chicken small intestinal epithelial cell (CSIEC) model to investigate the potential role of Bifidobacterium bifidum postbiotics (BbP) in preventing and treating S. Pullorum infections. S. Pullorum infection poses a significant challenge in the poultry industry due to its negative impact on intestinal health and immune responses (Liu et al., 2022; Zou et al., 2024), As an emerging alternative to antibiotics, BbP demonstrated remarkable effects in this study, including improving growth performance, modulating immunity, protecting intestinal barrier function, restoring gut microbial balance, and alleviating pyroptosis.

The growth performance of chicks was significantly improved by BbP, particularly after S. Pullorum infection. The body weight of chicks in the BbP+SP group was significantly higher than that in the SP group, indicating that B. bifidum postbiotics not only promoted growth before infection but also effectively alleviated the growth inhibition caused by S. Pullorum during the post-infection period. This is consistent with previous studies. For instance, Li et al. (2024) found that supplementing postbiotics derived from Bacillus subtilis improved growth performance, reduced mortality, and enhanced immunity and bone health in chicks (Li et al., 2024).Similarly, Chang et al. demonstrated that using fermentation products of lactic acid bacteria as feed additives improved body weight and feed conversion efficiency (Chang et al., 2022). Furthermore, BbP significantly reduced the mortality rate caused by S. Pullorum, with the BbP+SP group exhibiting the lowest mortality rate and showing strong protective effects.

Liver and intestinal pathological analyses further validated the protective effects of BbP. In the SP group, the liver exhibited significant pathological damage, including numerous white nodules on the surface, disorganized hepatocyte arrangement, sinusoidal dilation with congestion, fatty degeneration, and vacuolar degeneration, along with the formation of focal necrotic areas surrounded by inflammatory cell infiltration. In contrast, the BbP-treated group significantly alleviated liver damage. Particularly in the BbP+SP group, liver structures were almost d to normal, with no apparent necrotic areas and only mild inflammatory cell infiltration. Similarly, intestinal pathological results revealed severe tissue damage in the SP group, including villus shedding, epithelial necrosis and detachment, lamina propria edema, and capillary congestion in the duodenum, jejunum, ileum, and cecum. BbP treatment significantly reduced these pathological changes, with the BbP+SP group exhibiting the lowest lesion scores, and the intestinal tissues nearly restored to normal. Notably, BbP also restored villus length and crypt depth in the small intestine, bringing the villus-to-crypt ratio close to normal levels. Previous studies have reported that villus length is positively correlated with nutrient absorption capacity in chicks, while reduced crypt depth indicates intestinal atrophy or chronic damage, often associated with impaired intestinal function and reduced absorptive ability (Awad et al., 2009). These findings demonstrate that BbP could repair intestinal structures and promote nutrient absorption, further confirming its role in improving growth performance and alleviating S. Pullorum-induced damage.

Inflammatory factor levels in serum, organs, and intestines were also significantly modulated by BbP. In the SP group, serum levels of IL-1β and IL-8 were significantly elevated, while IL-10 and IL-4 were reduced. Similarly, mRNA expression of IL-1β, IL-8, Caspase-1, TLR4, and TNF-α in organs and intestines was significantly upregulated, while IL-10 mRNA expression was downregulated, indicating a strong pro-inflammatory response induced by S. Pullorum. In contrast, BbP exhibited stronger regulatory effects, enhancing anti-inflammatory factors and significantly suppressing pro-inflammatory responses, particularly in the BbP+SP group, which displayed the most pronounced immunosuppressive effects. These findings suggest that B. bifidum postbiotics alleviate excessive immune responses caused by S. Pullorum infection by restoring immune balance. This is consistent with the immunomodulatory effects of inactivated Lactobacillus plantarum and Lactobacillus rhamnosus in co-culture systems of human intestinal epithelial cells and macrophages (Magryś and Pawlik, 2023). Another key finding was the protective effect of BbP on intestinal barrier function. S. Pullorum infection significantly inhibited the expression of tight junction proteins such as ZO-1, Occludin, and Claudin-1 in the small intestine, likely contributing to intestinal barrier dysfunction. In contrast, BbP treatment significantly increased the mRNA expression of these tight junction proteins, with the BbP+SP group showing the most pronounced restoration of intestinal barrier function, outperforming live bacteria. This suggests that B. bifidum postbiotics enhance intestinal barrier integrity by repairing damaged barriers, effectively preventing the invasion and spread of S. Pullorum. These findings further support the protective role of postbiotics in maintaining intestinal health through enhanced barrier function and immune regulation (Liu et al., 2021; Zheng et al., 2023).

The colonization of S. Pullorum in the host intestine is a critical step in infection, and colonization levels directly affect the severity of the infection. S. Pullorum utilizes its type III secretion system to inject effector proteins into host cells. These proteins rearrange the host cell cytoskeleton, inducing membrane protrusions to engulf the bacteria and facilitate colonization (Ly and Casanova, 2007). Previous studies have demonstrated that host immune responses, intestinal barrier function, and gut microbiota diversity are key factors affecting S. Pullorum colonization (Valdez et al., 2009; Jacobson et al., 2018). Antibiotics disrupt the balance of gut microbiota, increasing the host's susceptibility to S. Pullorum infection(Sekirov et al., 2008). Moreover, antibiotics alter the composition of the microbiota, and even if the total bacterial count returns to normal, changes in specific bacterial populations may persist, leading to sustained susceptibility to S. Pullorum (Croswell et al., 2009). In this study, BbP treatment effectively modulated the gut microbiota. In the SP group, microbial diversity in the gut was significantly reduced, and the abundance of Proteobacteria increased, indicating S. Pullorum-induced dysbiosis (Liu et al., 2021). In contrast, the BbP+SP group significantly restored the abundance of Firmicutes and reduced the relative abundance of Proteobacteria, demonstrating the ability of B. bifidum postbiotics to modulate gut microbial composition. These findings align with other studies showing that probiotics can improve intestinal barrier function, alleviate inflammation, and correct microbial dysbiosis (Dimidi et al., 2017; Zheng et al., 2023). Furthermore, postbiotic intervention increased the relative abundance of beneficial bacteria such as Lachnospiraceae and Ruminococcaceae, while reducing the abundance of pathogenic bacteria such as Desulfovibrionaceae and Enterobacteriaceae, further demonstrating the role of postbiotics in restoring intestinal health. These findings support the potential application of BbP in poultry disease prevention, particularly as an antibiotic alternative.

In this study, we established a chicken small intestinal epithelial cell (CSIEC) model of Salmonella Pullorum infection to directly verify the ability of Bifidobacterium bifidum postbiotics (BbP) to enhance CSIEC cell viability and reduce S. Pullorum colonization. These findings are consistent with the animal experiments, where BbP significantly improved growth performance and reduced S. Pullorum colonization in various organs and the intestinal tract. We also systematically investigated the temporal changes in pyroptosis-related proteins induced by S. Pullorum.

First, S. Pullorum infection significantly upregulated pyroptosis markers, including ASC, Caspase-1, and GSDMD-N, with these changes observable as early as 3 h post-infection. ASC, as the core component of the inflammasome complex, is rapidly recruited during early infection to initiate Caspase-1 activation. At 3 h post-infection, the expression levels of ASC and Caspase-1 were significantly increased, closely correlating with inflammasome activation. Correspondingly, GSDMD-N expression was also significantly elevated, indicating that pyroptosis was already being triggered in the early stages of infection. These results align with previous studies, demonstrating that S. Pullorum activates pyroptosis via inflammasome activation, leading to cell membrane rupture and the release of cytokines and damage-associated molecular patterns (DAMPs), which exacerbate localized inflammatory responses (Clare, 2021; Pandeya et al., 2023). As the infection progressed (8 h, 13 h, and 20 h post-infection), the pyroptosis process became more pronounced. Our experimental results showed that the expression levels of ASC, Caspase-1, and GSDMD-N peaked at 8 h and remained elevated at 13 and 20 h. During this process, the N-terminal domain of GSDMD was cleaved and formed pores in the cell membrane, leading to the release of intracellular contents, including cytokines such as IL-1β and IL-18. These cytokines further activated localized immune responses and intensified inflammation (Liu et al., 2024). This indicates that pyroptosis, while an early defensive response during S. Pullorum infection, can exacerbate tissue damage when excessively activated in later stages. Interestingly, when BbP was used as a pretreatment, the activation of pyroptosis was significantly suppressed. By comparing the changes in indicators across the groups, we found that at 3 h post-infection, the expression levels of ASC, Caspase-1, and GSDMD-N in the BbP+SP group were significantly lower than those in the SP group (*P* < 0.05). This inhibitory effect persisted at 8 h, 13 h, and 20 h post-infection. These results indicate that BbP reduces the extent of pyroptosis by inhibiting inflammasome activation, thereby alleviating the immune hyperactivation and tissue damage caused by S. Pullorum infection.

In conclusion, *Bifidobacterium bifidum* postbiotics (BbP) demonstrated significant efficacy in preventing *S. Pullorum* infection by improving growth performance, alleviating immune damage, and restoring intestinal barrier function. BbP regulated immune responses, restored gut microbial balance, and enhanced intestinal barrier integrity, demonstrating its potential as an anti-infective agent. Furthermore, BbP inhibited pyroptosis and inflammation, providing further protection to the intestinal barrier and modulating the gut microbiota. These findings provide critical experimental evidence supporting the application of BbP in poultry disease prevention, particularly as an antibiotic alternative.

However, certain limitations of this study should be acknowledged. The use of a standard laboratory strain of *S. Pullorum* (CVCC 533) may not fully capture the genetic and phenotypic diversity of clinical isolates. Future studies should include multiple *S. Pullorum* strains isolated from field outbreaks to evaluate the broader applicability of BbP in real-world poultry farming scenarios. Additionally, further research is needed to explore the long-term effects of postbiotic administration and its potential interactions with other feed additives or vaccines. These investigations will help optimize postbiotic formulations and application strategies for practical use in the poultry industry. Overall, this study lays a solid foundation for the development of postbiotics as a sustainable and effective alternative to antibiotics in poultry production.

## Declaration of competing interest

The authors declare that they have no known competing financial interests or personal relationships that could have appeared to influence the work reported in this paper.

## Appendix Supplementary materials

Image, application 1
