# Supplementary material for: Bifidobacterium bifidum postbiotics prevent Salmonella Pullorum infection in chickens by modulating pyroptosis and enhancing gut health
Source: Poult Sci. 2025 Mar 1;104(4):104968. doi: 10.1016/j.psj.2025.104968 (PMC11927735; doi:10.1016/j.psj.2025.104968)
Supplement: Supplementary file 1 [file mmc1.docx]

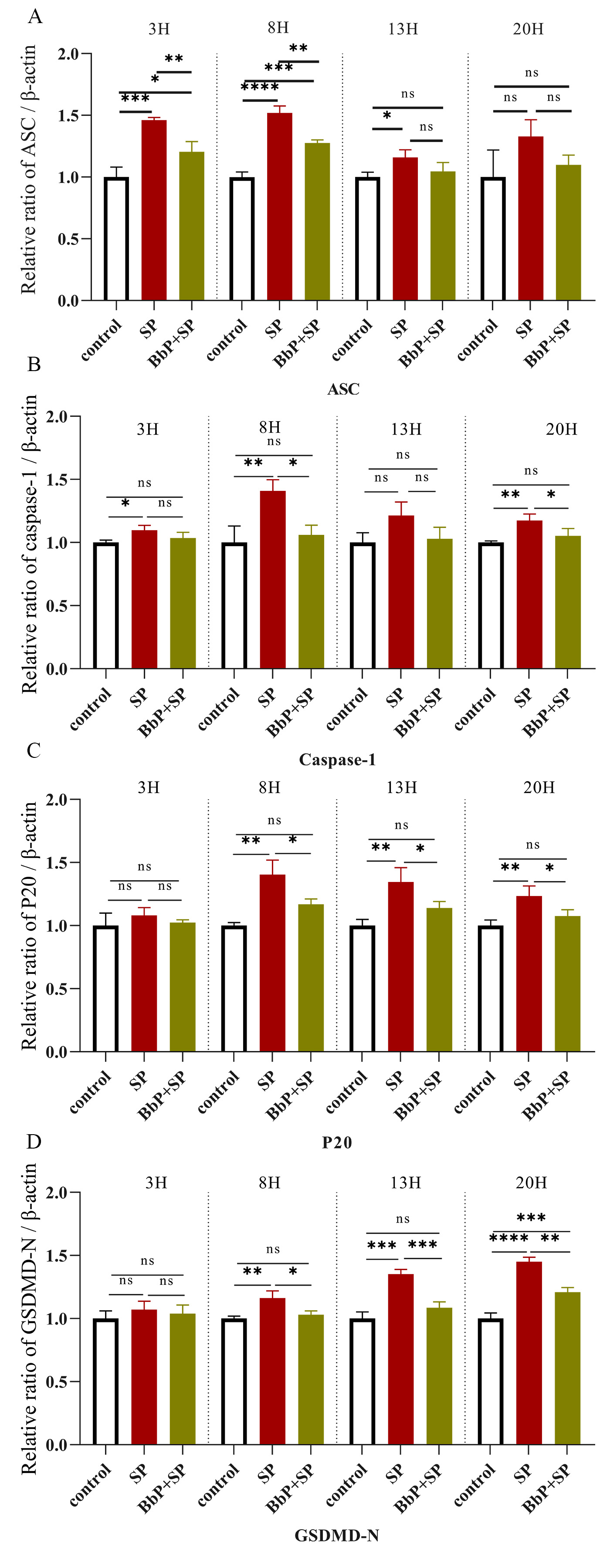


Supplementary Figure. S1. Gray value analysis of pyroptosis-related protein expression in chicken small intestinal epithelial cells (CSIEC).

(A) Normalized gray values for ASC expression at 3 h, 8 h, 13 h, and 20 h post-infection; (B) Normalized gray values for Caspase-1 expression at 3 h, 8 h, 13 h, and 20 h post-infection; (C) Normalized gray values for the activated fragment of Caspase-1 (P20) at 3 h, 8 h, 13 h, and 20 h post-infection; (D) Normalized gray values for GSDMD-N expression at 3 h, 8 h, 13 h, and 20 h post-infection. Data are presented as Mean ± SD (n = 3). Statistical significance was determined by one-way ANOVA followed by Tukey’s test. *P < 0.05, **P < 0.01, ***and****P<0.001.
